# Zoledronic Acid Enhances Lipopolysaccharide-Stimulated Proinflammatory Reactions through Controlled Expression of SOCS1 in Macrophages

**DOI:** 10.1371/journal.pone.0067906

**Published:** 2013-07-09

**Authors:** Daichi Muratsu, Daigo Yoshiga, Takaharu Taketomi, Tomohiro Onimura, Yoshihiro Seki, Akinobu Matsumoto, Seiji Nakamura

**Affiliations:** 1 Section of Oral and Maxillofacial Oncology, Division of Maxillofacial Diagnostic and Surgical Sciences, Faculty of Dental Science, Kyushu University, Fukuoka, Japan; 2 Division of Oral and Maxillofacial Reconstructive Surgery, Department of Oral and Maxillofacial Surgery, Kyushu Dental College, Fukuoka, Japan; 3 Department of Neuropsychiatry, Graduate School of Medical Sciences, Kyushu University, Fukuoka, Japan; 4 Department of Molecular and Cellular Biology, Medical Institute of Bioregulation, Kyushu University, Fukuoka, Japan; 5 Beth Israel Deaconess Cancer Center; Cancer Genetics Program and Division of Genetics, Department of Medicine, Beth Israel Deaconess Medical Center; and Departments of Medicine and Pathology, Harvard Medical School, Boston, Massachusetts, United States of America; University of Florida, College of Dentistry & The Emerging Pathogens Institute, United States of America

## Abstract

Bisphosphonate-related osteonecrosis of the jaw (BRONJ) is a serious side effect of nitrogen-containing bisphosphonate (NBP) use. Many studies have shown that BRONJ is limited to the jawbone and does not occur in the other bones. We hypothesized that BRONJ is related to local bacterial iections and involves the innate immune system. To examine the relationship between BRONJ and innate immunity, we examined the effects of NBPs on macrophages, one of the important cell types in innate immunity. The expression of toll-like receptor-4 (TLR4) in cells after pretreatment with zoledronic acid (ZOL) did not considerably differ from that in untreated control cells. However, cytokine levels and nitric oxide (NO) production increased after pretreatment with ZOL. Furthermore, ZOL induced NF-κB activation by enhancing IκB-α degradation. Lipopolysaccharide (LPS)-induced apoptosis also increased after pretreatment with ZOL. This effect was mediated by a reduction of suppressor of cytokine signaling-1 (SOCS1), which is a negative regulator of myeloid differentiation primary response gene 88 (MyD 88)-dependent signaling. These results suggest that ZOL induced excessive innate immune response and proinflammatory cytokine production and that these processes may be involved in the bone destruction observed in BRONJ.

## Introduction

Nitrogen-containing bisphosphonates (NBPs), which are synthetic analogs of pyrophosphate, are an effective treatment for osteoporosis, hypercalcemia of malignancy, osteolytic lesions in multiple myeloma, and bone metastases from solid tumors, including breast cancer and hormone-independent prostate cancer [1], [2]. In addition, NBPs are effective for the treatment of numerous metabolic bone diseases. NBPs have a high affinity for bone minerals [3] and accumulate in high concentrations in bones [4], [5]. They are selectively taken up by osteoclasts and strongly inhibit bone resorption by inducing apoptosis in osteoclasts [6], [7].

However, serious side effects such as bisphosphonate-related osteonecrosis of the jaw (BRONJ) have been reported with the use of NBPs [8], [9]. In addition to the clinical appearance of exposed necrotic bone, concomitant local infections are often observed in patients with BRONJ. It has been reported that bisphosphonate-related osteonecrosis is limited to the jawbone and is not observed in other bones. In afflicted patients, BRONJ adversely affects quality of life and produces significant morbidity. Because bisphosphonates have been shown to reduce bone remodeling [10] and angiogenesis [11], the suppression of bone turnover and jaw angiogenesis resulting from bisphosphonates have been proposed as underlying mechanisms for BRONJ.

Myriad numbers of bacteria reside in the mouth. Therefore, it has been suggested that infection triggers the development of BRONJ. Infections of the jawbone cause inflammation, which intensifies as the severity of BRONJ increases [12]. Innate immunity has a critical role in the inflammation process, and innate immune surveillance relies in part on the recognition of conserved molecules unique to some classes of potential pathogens. For example, bacterial lipopolysaccharide (LPS), which is found in the cell wall of Gram-negative bacteria, is a potent inducer of immune responses. Toll-like receptors (TLRs), originally identified as key mediators of development in Drosophila [13], initiate second-messenger pathways that regulate the expression of genes required for protective immune responses. For example, activation through TLRs has been shown to induce potent inflammatory responses, including the production of reactive oxygen and nitrogen intermediates, the secretion of chemokines and cytokines, and cellular differentiation, and many of these responses are regulated by NF-κB [14]. Each TLR has a specific ligand that binds and activates it.

Depending on the oral cavity environment, the oral flora changes in various ways. For example, when the oral cavity environment gets worse, gingival inflammation occurs. As a result, the proportion of Gram-negative bacteria producing LPS increase. LPS-induced inflammatory cytokines specifically bind to TLR4. Therefore, in this study, we used macrophages, which are of the same origin as osteoclasts, like all typical immune cells, and we focused on the relationship between innate immunity and BRONJ, specifically the involvement of the NBP zoledronic acid (ZOL), and its effects on TLR4 signaling and inflammatory cytokine production.

## Materials and Methods

### Cell Culture

The murine macrophage cell line, RAW264.7, was obtained from the Division of Host Defense, Research Center of Prevention of Infectious Disease, Medical Institute of Bioregulation, Kyushu University [15] and maintained in Dulbecco’s modified Eagle’s medium (DMEM) (Sigma Aldrich, USA) supplemented with 10% decomplemented fetal bovine serum, 2 mM glutamine, 1 mM sodium pyruvate, 100 U/mL penicillin, and 100 µg/mL streptomycin (Invitrogen, USA) in a humidified atmosphere containing 5% CO_2_ at 37°C.

### Reagents

ZOL was purchased from Novartis Pharma AG (Switzerland) as a hydrated disodium salt. *Escherichia coli* 0111: B4 LPS was purchased from Sigma (UK).

### Real-time Reverse Transcription-polymerase Chain Reaction (RT-PCR)

RNA was extracted from RAW264.7 cells using TRIzol Reagent (Invitrogen, USA) as according to the manufacturer’s protocol. First-strand complementary DNA (cDNA) was synthesized from total RNA (1.0 µg) in a final volume of 20 µL according to the Reverse Transcriptase cDNA synthesis protocol. Quantitative real-time PCR was performed with Brilliant III Ultra-Fast SYBR® Green QRT-PCR Master Mix (Agilent Technologies, USA). using the following gene-specific primers: *mouse-IL-1β*: forward, CAGGATGAGGACATGAGCACC; reverse, CTCTGCAGACTCAAACTCCAC; *mouse-IL-6*: forward, CCAGAGATACAAAGAAATGATGG; reverse, ACTCCAGAAGACCAGAGGAAT; *mouse-TNF-α*: forward, GGGGCCACCACGCTCTTCTG; reverse, GGCAGGGGCTCTTGACGGC; *mouse-iNOS*: forward, CCCTTCCGAAGTTTCTGGCAGCAG; reverse, GGCTGTCAGAGCCTCGTGGCTTTGG; and *mouse-*glyceraldehyde 3-phosphate dehydrogenase (*GAPDH*): forward, CAATGCATCCTGCACCACCAA; reverse, GTCATTGAGAGCAATGCCAG. The specificities of the expected products were demonstrated by melting curve analyses. GAPDH was used as an internal standard for mRNA analysis. PCR reactions for each sample were performed in triplicate. The real-time PCR data were quantified by the ΔCT method using the following formula: n = 100^*2^ - (ΔCT targeted gene - ΔCT *GAPDH*).

### Quantification of Nitric Oxide (NO) Release

The accumulation of NO_2_^-^, a stable end product extensively used as an indicator of NO production by cultured cells, was assayed using the Griess reaction kit (Dojindo Laboratories, Japan) according to the manufacturer’s instructions. RAW264.7 cells were plated in 96-well tissue culture plates at a density of 2 × 10^3^ cells per well (in 200 µL), and preincubated with or without ZOL (10 µL) for 24 h, and then incubated with LPS (100 ng/mL) at 37°C for another 24 h. The cell-free supernatants were then mixed with equal amounts of Griess reagent and incubated at room temperature for 15 min, and then the absorbance at 540 nm was read using a plate reader (Labsystems Multiscan MS, Germany).

### Flow Cytometry

RAW264.7 cells were plated in 6-well tissue culture plates at a density of 2 × 10^3^ cells per well in DMEM with 10% fetal bovine serum. After 24 h of culture, cells were incubated in the presence or absence of 10 µM ZOL with or without 100 ng/mL LPS at 37°C for an additional 24, 48, or 72 h. Following bisphosphonate treatment, RAW264.7 cells were harvested with 0.25% trypsin-EDTA and then washed 3 times in phosphate-buffered saline. Combined cell pools were finally resuspended in 250 mL of labeling solution according to the manufacturer’s instructions and then incubated for 10–15 min in the dark. Labeled cells were then counted in a flow cytometer (FACS Verse™; BD Biosciences, USA) within 30 min. Annexin-V-fluorescein isothiocyanate (FITC) labeling was measured at 518 nm in the FL-1 channel (FITC detector), and propidium iodide (PI) staining was measured at 620 nm in the FL-2-channel (phycoerythrin detector).

### Western Blotting

RAW264.7 cells were incubated with ZOL in DMEM and 10% fetal bovine serum for 24 h. After harvesting, whole cell lysates were prepared by washing the cells 3 times with phosphate-buffered saline and then resuspending them in lysis buffer (10 mM Tris-HCl [pH 7.6], 150 mM NaCl, 1% Triton X-100, and a cocktail of protease inhibitors; Boehringer Mannheim, Switzerland). Insoluble material was removed by centrifugation at 12,000 rpm for 10 min at 4°C. Samples containing equal protein were mixed with 5× sample buffer (20% glycerol, 10% 0-mercaptoethanol, 6% sodium dodecyl sulfate, and 125mM Tris-HCl [pH 6.8]). Protein samples were separated with 10% sodium dodecyl sulfate-polyacrylamide gel electrophoresis and then transferred to polyvinyl difluoride membranes (Immobilon-P; Millipore, USA). Membranes were blocked with blocking solution (50 mM Tris-HCl, 150 mM NaCl, nonfat dry milk, and 0.1% Tween) for 30 min at room temperature and then incubated overnight with anti-phospho- IκB-α (#9246; Cell Signaling, USA), anti- IκB-α (#9242; Cell Signaling, USA), anti-signal transducer activator of transcription 1 (STAT1, #9172; Cell Signaling, USA), anti-phospho-STAT1 (#9171; Cell Signaling, USA), anti-suppressor of cytokine signaling 1 (SOCS1, ab65989; Abcam, USA), anti-myeloid differentiation primary response gene 88 (MyD88, ab2068; Abcam, USA), or anti-extracellular signal-regulated kinase 2 (ERK2, #12607; Santa Cruz, USA) primary antibodies at 4°C. The membranes were washed thoroughly with washing buffer (0.32 M sucrose, 10 mM HEPES, and 0.1 mM EDTA [pH 7.4]) and incubated with anti-rabbit IgG at a 1∶10,000 dilution for 30 min. The proteins were visualized with SuperSignal West Pico Chemiluminescent Substrate (Thermo Fisher Scientific, USA) or Immobilon Western Detection Reagents (Millipore, Germany).

## Results

### Effects of ZOL Pretreatment on LPS-induced Expression of Cytokines

**Figure 1:**
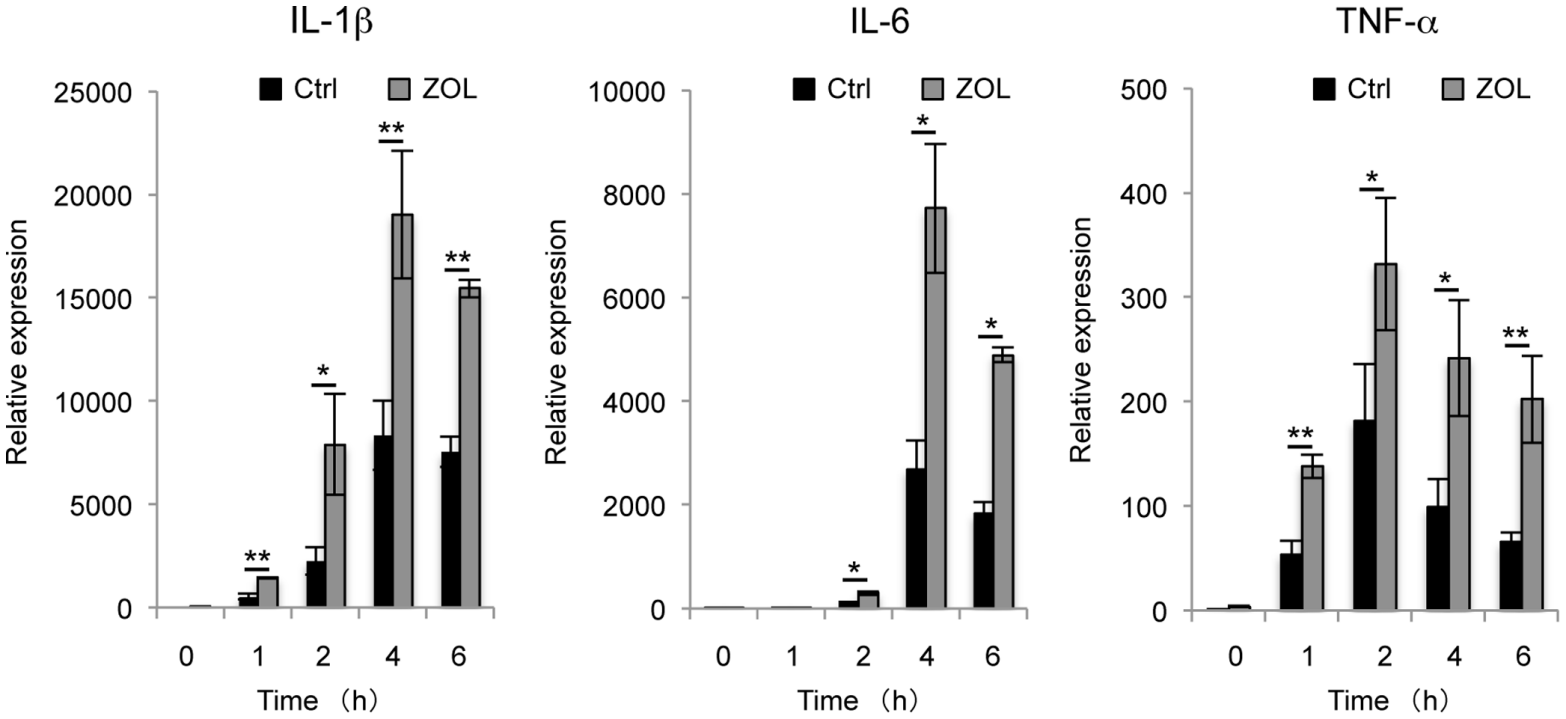
Effects of ZOL pretreatment on LPS-induced cytokine expression. RAW264.7 cells were cultured with or without ZOL for 24 h and then with LPS for an additional 0, 1, 2, 4, or 6 h. After treatment, the expression of *IL-1β*, *IL-6,* and *TNF-α* was examined by real-time PCR. LPS-stimulated inflammatory cytokine (*IL-1β*, *IL-6*, and *TNF-α*) production by RAW264.7 cells increased significantly after ZOL pretreatment. Significant differences from the negative controls that were not treated with ZOL are indicated as follows: *P<0.05 and **P<0.01.

*Interleukin (IL)-1β*, *IL-6*, and *tumor necrosis factor (TNF)-α* are important inflammatory cytokines. Therefore, we examined the effects of ZOL on LPS-induced release of *IL-1β*, *IL-6*, and *TNF-α* from RAW264.7 cells. RAW264.7 cells were pretreated with ZOL (10 µM) for 24 h and then incubated with LPS (100 ng/mL) for 0, 1, 2, 4, or 6 h. LPS-stimulated production of *IL-1β*, *IL-6*, and *TNF-α* from RAW264.7 cells increased significantly after ZOL pretreatment (Figure 1).

### Effects of ZOL on NO Release from LPS-stimulated Macrophages

**Figure 2:**
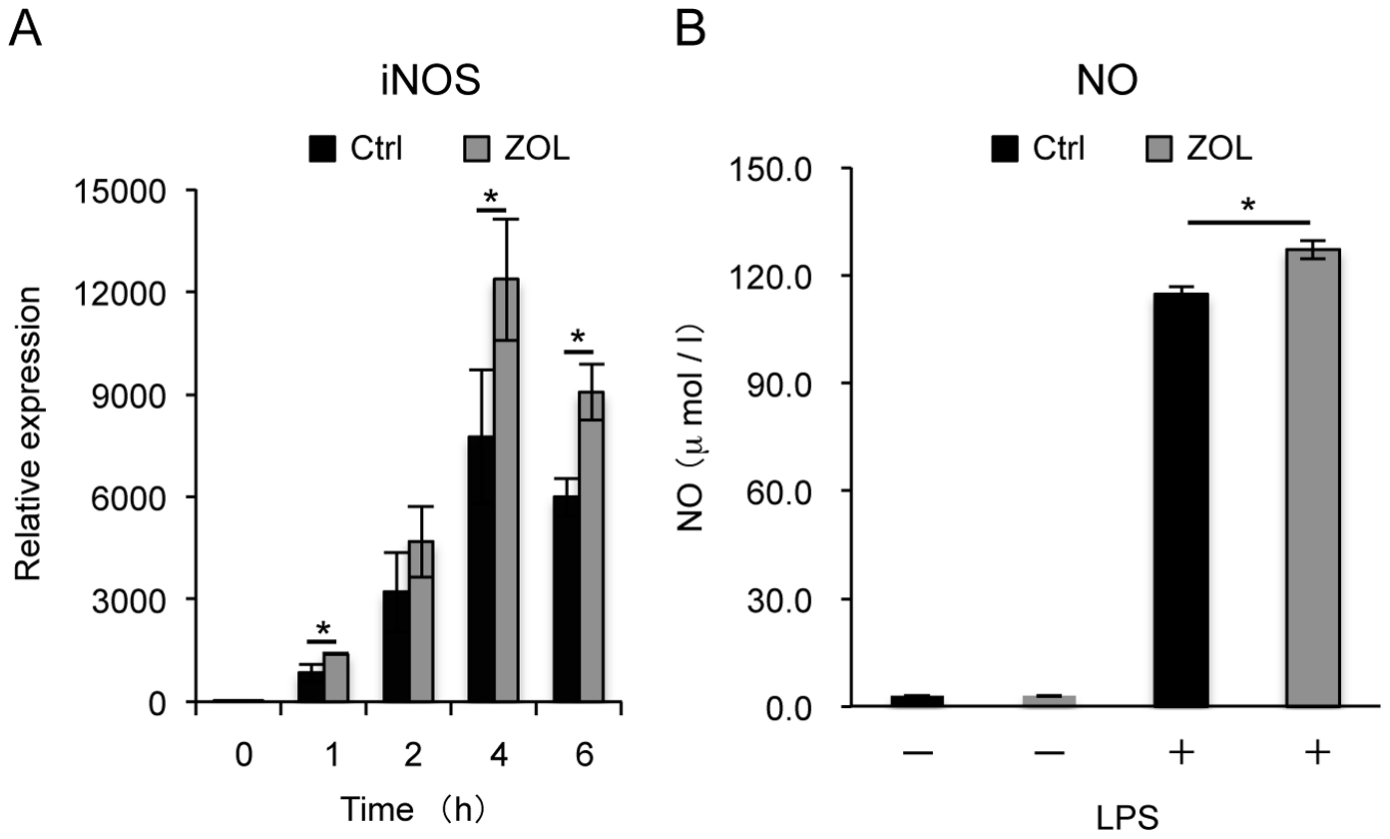
Effects of ZOL on NO release from LPS-stimulated macrophages. RAW264.7 cells were cultured with or without ZOL for 24 h and then with LPS for an additional 0, 1, 2, 4, or 6 h. After treatment, iNOS expression was examined by real-time PCR. LPS-stimulated production of iNOS from RAW264.7 cells increased significantly after ZOL pretreatment. Significant differences from the negative controls that were not treated with ZOL are indicated by an asterisk (*P<0.05). (B) RAW264.7 cells were cultured with or without ZOL (10 µM) and LPS (100 ng/mL) for 24 h. NO release was measured by the Griess method. NO production was significantly higher in ZOL-pretreated cells than in LPS-treated positive controls. ZOL had no effect on the release of NO. Significant differences from the negative controls that were not treated with ZOL are indicated by an asterisk (*P<0.05).

NO is an index of inflammation that is generated by iNOS. We examined the effects of ZOL on LPS-induced iNOS release from RAW264.7 cells. RAW264.7 cells were pretreated with ZOL (10 µM) for 24 h and then incubated with LPS (100 ng/mL) for 0, 1, 2, 4, or 6 h. LPS-stimulated production of iNOS by RAW264.7 cells increased significantly after ZOL pretreatment (Figure 2A). RAW264.7 cells were pretreated with ZOL for 24 h, and then treated with LPS in the presence of ZOL for 24 h. NO production was significantly higher in ZOL-pretreated cells than in the LPS-treated controls, suggesting that ZOL accelerates NO release (Figure 2B). ZOL had no effect on the release of NO (Figure 2B).

### Effects of ZOL on LPS-induced Cell Apoptosis

**Figure 3:**
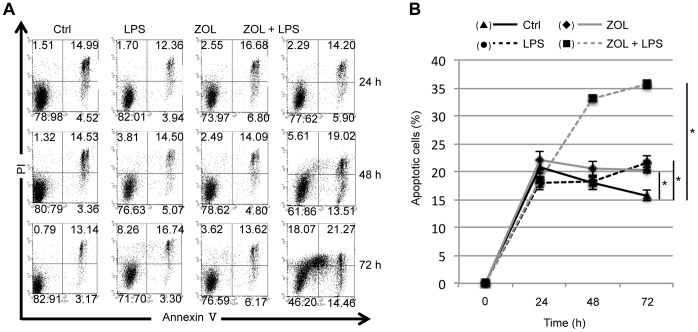
Effects of ZOL on LPS-induced cell apoptosis. RAW264.7 cells were incubated for the indicated times (0, 24, 48, or 72 h) with control medium, LPS (100 ng/mL), ZOL (10 µM), or ZOL+LPS. annexin V and PI were added to the cultures prior to flow cytometry. See the methods for a detailed explanation of the contour plots. (B) Plot of apoptosis in (▴) controls, (•) LPS-treated, (□) ZOL-treated, and (▪) ZOL+LPS-treated cells. After stimulation with LPS, apoptosis increased compared to that in unstimulated controls. A similar amount of LPS-stimulated apoptosis was observed after ZOL pretreatment, and a significant increase in apoptosis was observed after further LPS stimulation. Significant differences from the cikb ontrols that were not treated with ZOL are indicated by an asterisk (*P<0.01).

Cells were stained with annexin-V-FITC and PI, and the results are presented as contour plots of PI versus annexin-V staining intensity (Figure 3A). The cells represented in the 4 quadrants of these plots are as follows. The lower left includes cells that stained negatively for both annexin-V and PI, and these were considered undamaged cells. The lower right shows PI-negative cells with moderate annexin-V staining, and these were considered early apoptotic cells. The upper left includes annexin-V-negative cells with high PI staining, and these were classified as necrotic cells. The upper right includes cells that were both annexin-V and PI-positive, and these were considered late apoptotic cells. These results are expressed as the percentage of positively-stained RAW264.7 cells (Figure 3A). The controls exhibited increased apoptosis at 24 h followed by decreased apoptosis. After stimulation with LPS, apoptosis increased compared to that of the controls. The same degree of apoptosis was observed after LPS stimulation with ZOL pretreatment, whereas a significant increase in apoptosis was observed after further LPS stimulation (Figure 3B).

### Effects of ZOL on LPS-induced TLR4-mediated Activation of NF-κB

Translocation of NF-κB to the nucleus and its effects on inflammatory genes are preceded by the phosphorylation, ubiquitination, and proteasome degradation of IκB-α.

**Figure 4:**
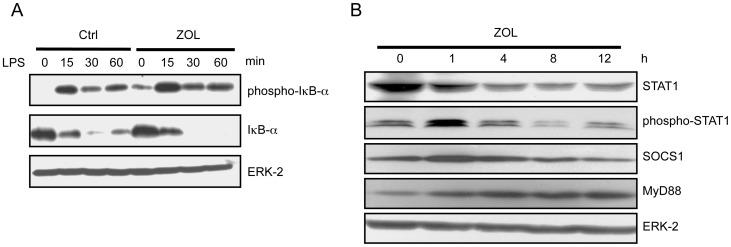
Effects of ZOL on LPS-induced TLR4-mediated activation of NF-κB cytokines. (A) RAW264.7 cells were cultured with or without ZOL (10 µM) for 24 h and then with LPS (100 ng/mL) for the indicated times (0, 15, 30, or 60 min). The levels of phospho-IκB-α, IκB-α, and ERK2 were analyzed by western blotting. These data indicated that ZOL treatment increased the levels of phosphorylated IκB-α and enhanced the degradation of IκB-α. (B) RAW264.7 cells were cultured with ZOL for the indicated times (0, 1, 4, 8, or 12 h). STAT1 protein levels decreased over time after ZOL treatment. The levels of Phospho-STAT1 increased 1 h after ZOL treatment and then decreased in a time-dependent manner. The levels of SOCS1, which suppresses TLR4 signaling, increased 1 h after ZOL treatment and then decreased in a time-dependent manner. The levels of MyD88 protein increased with time after ZOL treatment.

To determine whether ZOL has an effect on IκB-α phosphorylation and its subsequent degradation, we examined the levels of phospho-IκB-α and total IκB-α expression by western blotting. RAW264.7 cells were pretreated with ZOL (10 µM) for 24 h and then incubated with LPS (100 ng/mL) for 0, 15, 30, or 60 min. After incubation, RAW264.7 cells were lysed and assayed by western blotting using antibodies against phosphorylated IκB-α and total IκB-α. These data indicated that ZOL treatment increased the levels of phosphorylated IκB-α and enhanced the degradation of IκB-α (Figure 4A).

TLR4 signaling is divided into MyD88-dependent and MyD88-independent (Toll/IL-1 receptor-domain containing adaptor-inducing and IFN-β-dependent) pathways. The MyD88-dependent pathway mediates the expression of proinflammatory cytokines, whereas the MyD88-independent pathway mediates the induction of type-1 interferons and interferon-inducible genes. TLR4 stimulation can induce potent responses, which explains why inhibitory pathways are necessary to protect the host from inflammation-induced damage. Some reports have suggested that TLR4 signaling is regulated at multiple levels by numerous negative regulators [16]. One negative regulator, SOCS1, induces ubiquitination of the Toll/IL-1 receptor domain containing adaptor protein, which is upstream of MyD88, and its subsequent degradation. STAT1 is phosphorylated by Janus kinase/STAT signaling, and induces SOCS1. SOCS1 is a negative feedback regulator of signaling molecules such as LPS, and the induction of SOCS1 expression can decrease macrophage cytokine production [17]. In our studies, STAT1 protein levels decreased over time after ZOL treatment. The levels of Phospho-STAT1 increased 1 h after ZOL treatment and then decreased in a time-dependent manner. Therefore, the levels of SOCS1, whose expression is induced by Phospho-STAT1, increased 1 h after ZOL treatment, and then decreased in a time-dependent manner. MyD88 protein levels increased over time after ZOL treatment (Figure 4B). These results indicate that ZOL might be important for regulating cellular SOCS1 accumulation.

## Discussion

The accumulation of NBPs, which can decrease bone metabolism, does not properly induce tissue repair that normally occurs in response to an induced or a physiological trauma, leading to the exposure of necrotic bone to the oral environment [11], [18], [19].

A proposed hypothesis for the development of BRONJ is an alteration in bone turnover associated with the particular characteristics of the jaw bone [10], [20], such as mucosal coating, frequent risk of infection, and constant potential for trauma [18], [21]. Some authors have described the appearance of BRONJ along with *Actinomyces* infections and have reported several cases involving bone necrosis and osteomyelitis that were caused by this microorganism [22].

However, there have been few reports on bacterial infection and innate immunity in association with BRONJ. In our study, the influence of ZOL, a NBP, on TLR4 signaling in RAW264.7 cells was examined.

LPS binding to TLR4 induces inflammatory cytokines and nitric oxide by activating NF-κB because intracellular signal transduction through various TLR4 downstream adaptor molecules occurs, resulting in the production of nitric oxide synthase.

We showed that LPS-stimulated induction of inflammatory cytokines IL-1β, IL-6, and TNF-α and NO production in RAW264.7 cells were enhanced after pretreatment with ZOL. Although inflammatory cytokines in blood, such as TNF-α and IL-6, are reportedly increased in patients treated with NBPs [23], we did not find any significant increase in these inflammatory cytokines in RAW264.7 cells after stimulation with ZOL alone.

Inflammation involves multiple cascades mediated by activated inflammatory or immune cells. In these cascades, a number of immunopathological changes occur, including the overproduction of NO, proinflammatory cytokines such as IL-1β, IL-6, and TNF-α, and other detrimental mediators, including caspases, that in turn activate apoptosis [24], [25].

TNF-α overproduction stimulates the generation of other pro-oxidant mediators that directly induce cell injury. Among these, free radicals such as NO seem to play a central role [26]. The inflammatory cytokines IL-1β and TNF-α are involved in the induction of cell apoptosis. IL-1β is produced as an inactive precursor that assumes the active form through excision of a precursor domain by an IL-1β-converting enzyme. This enzyme, caspase 1, is the first caspase discovered and it is among a group of cysteine proteases that govern the apoptotic process. TNF-α acts as an apoptosis-inducing factor by activating caspase 8 through adaptor molecules such as the TNF receptor type 1-associated death domain molecule and the Fas-associated death domain molecule [27]. It is known that NBPs induce cellular apoptosis by inhibiting the mevalonic acid pathway [28], [29]. In the present study, apoptosis was increased by the administration of ZOL. Furthermore, apoptosis in the ZOL-treated cells was highly accelerated by stimulation with LPS. The inhibition of mevalonic acid pathway by ZOL and the hypersecretion of inflammatory cytokines in the ZOL-treated cells upon stimulation with LPS were considered as the reason that apoptosis increased.

In TLR signal transduction, IκB-α is bound to NF-κB, which is directly involved in the production of inflammatory cytokines; thus, it acts as a suppressive factor [30]. Phosphorylation of IκB-α at Ser 32 triggers its ubiquitination and subsequent proteolysis via the proteasome pathway. NF-κB then translocates into the nucleus and then inflammatory cytokines are expressed. In the present study, inflammatory cytokine expression in ZOL-treated cells was enhanced by increased translocation of NF-κB into the nucleus due to elevated LPS-stimulated IκB-α phosphorylation and accelerated proteolysis. IL-1 receptor-associated kinase M (IRAK-M) and SOCS1 are known suppressors of this signal, and they both inhibit the enzymatic activity of IRAK [17]. IRAK-M is specifically expressed in monocytes and macrophages and is induced through the NF-κB pathway [31]. IRAK-M inhibits the formation of the IRAK-TRAF6 complex by suppressing the dissociation of IRAK-1 and IRAK-4 from MyD88. SOCS1, a cytokine-inducing protein with an SH2 domain, is induced by TLR stimulation and inhibits the activation of NF-κB and STAT1. It has been reported that, in the absence of SOCS1, the inflammatory signals of IFN-γ are excessively activated, resulting in the disappearance of the suppressive effects of prostaglandin E2 (PGE2) [32]. Therefore, excessive inflammation might be provoked by suppression of the anti-inflammatory actions of elements such as IRAK-M and SOCS1, and an excessive inflammatory condition might develop due to suppression of the anti-inflammatory PGE2. It has also been reported that IRAK-M and SOCS3 are attenuated by NBPs [33], [34]. In our study, SOCS1 expression was attenuated by ZOL alone, which was similar to the results found with SOCS3. In addition, MyD88 expression increased, which was thought to be a result of attenuated SOCS1 and IRAK-M expression due to stimulation with BPs that downregulated their activation as part of a negative feedback mechanism.

The results of this study have led us to speculate the mechanism of BRONJ. BRONJ onset is thought to be associated with delay of the physiological remodeling speed of the jawbone, the suppression of angiogenesis, delayed wound healing, and local bacterial infections [35]. NBPs accumulate as bone is metabolized and their potent suppression of osteoclasts leads to metabolic suppression in bones throughout the body [4], [5]. The concentration of NBPs is selectively increased in bones where metabolism is naturally active. The jawbone and in particular the alveolar bone, which is a dental-supporting tissue, are constantly exposed to the strong masticatory pressure that accompanies eating. For this reason, the remodeling of the alveolar bone is faster than other bones in the body. Due to the high rate of bone metabolism, NBPs selectively accumulate in high concentrations in the alveolar bone area. In addition, because the oral cavity has abundant bacterial flora, it is easy to cause infection. Therefore, bone infections occur as the bone becomes exposed due to surgical procedures and wounds. Then, the high concentrations of NBPs are diffused from the alveolar bone. Diffuse NBPs affects immune cells that have been induced by the infection. As a result, immune cells produce large amounts of inflammatory cytokines to give rise to a hyperinflammatory state. In the affected area, diffuse NBPs affects fibroblasts and causes delayed wound healing [35], further infection and aggravation of inflammation, which continues to be repeated, extending the osteonecrosis. In the present study, the inhibitory effects of ZOL on SOCS1 expression were first confirmed in RAW264.7 cells. In conclusion, our data strongly suggest that onset mechanism of BRONJ is due to the dysregulation of the inflammatory cytokine output with ZOL.
